# Sparse canonical correlation analysis for identifying, connecting and completing gene-expression networks

**DOI:** 10.1186/1471-2105-10-315

**Published:** 2009-09-28

**Authors:** Sandra Waaijenborg, Aeilko H Zwinderman

**Affiliations:** 1 Clinical Epidemiology, Biostatistics & Bioinformatics, Academic Medical Center, University of Amsterdam, PO Box 22700, 1100 DE Amsterdam, the Netherlands

## Abstract

**Background:**

We generalized penalized canonical correlation analysis for analyzing microarray gene-expression measurements for checking completeness of known metabolic pathways and identifying candidate genes for incorporation in the pathway. We used Wold's method for calculation of the canonical variates, and we applied ridge penalization to the regression of pathway genes on canonical variates of the non-pathway genes, and the elastic net to the regression of non-pathway genes on the canonical variates of the pathway genes.

**Results:**

We performed a small simulation to illustrate the model's capability to identify new candidate genes to incorporate in the pathway: in our simulations it appeared that a gene was correctly identified if the correlation with the pathway genes was 0.3 or more. We applied the methods to a gene-expression microarray data set of 12, 209 genes measured in 45 patients with glioblastoma, and we considered genes to incorporate in the glioma-pathway: we identified more than 25 genes that correlated > 0.9 with canonical variates of the pathway genes.

**Conclusion:**

We concluded that penalized canonical correlation analysis is a powerful tool to identify candidate genes in pathway analysis.

## Background

A molecular genetic pathway is a hypothesis or model on how the expression of different genes in a series of biochemical relationships influence each other and eventually lead to a specific phenotypical expression [1]. A reconstruction of a pathway breaks down metabolism pathways into their respective reactions and enzymes, and analyzes them within the perspective of the entire network. In simplified terms, a reconstruction involves collecting all of the relevant metabolic information of an organism and then compiling it in a way that makes sense for various types of analyses to be performed. The correlation between the genome and metabolism is made by searching gene databases, such as KEGG [2], GeneDB [3], etc., for particular genes by inputting enzyme or protein names [4]. Validity of pathways are often tested by controlled experiments, for instance by knocking-out or by overstimulation of specific genes, and then comparing the observed changes of enzymes and metabolites to what was predicted on the basis of the pathway.

Few pathways are thoroughly established, and many pathways are incomplete [5]. Since knock-out experiments are extremely expensive and time-consuming, genome wide gene-, protein-, and metabolite-expression studies are used for searching for genes, enzymes, and proteins that have a specific function in pathways of particular interest [6,7]. Pathways vary in size but usually contain a limited number of genes or enzymes, say up to a few hundreds, or thousands for middle-sized pathways [2]. When in a genome wide expression study microarrays are used to find new genes, then there might be easily tens of thousands of new candidates, causing a huge statistical multiple testing problem.

Recently we and others developed penalized canonical correlation analysis (PCCA) to quantify the association between two sets of genomic data [8,9]. We now generalized PCCA to identify genes/enzymes from a large set of candidates to complement the set of genes comprising a hypothesized pathway. The analysis is based on the availability of expression data of genes in a specific pathway measured in a sample of patients and the availability of expression data of a large set of candidate genes measured in the same samples. In this paper we will first describe PCCA. Next we will discuss different penalties which are needed to make the inference feasible, and how to estimate optimal values for the penalty-parameters involved. With a few simulations we will illustrate that our method is capable of identifying the correct genes. Finally, we will apply our methods on assessing the glioma-pathway [2] in 45 samples from patients with glioblastoma.

## Methods

### Penalized Canonical Correlation Analysis

Our objective is to extract groups of variables that capture common features out of two sets of variables, one containing information about expression of genes known to be in the same pathway and one containing expression of genes, some of which are candidates to be part of the same pathway. Consider the *n *× *m *matrix **Y**, containing *m *(gene expression) variables and the *n *× *k *matrix **X**, containing *k *variables, obtained from *n *individuals. Canonical correlation analysis (CCA) seeks for linear combinations of all the variables in **Y **which correlate maximally with linear combinations of all the variables in **X**. These linear combinations are the so-called canonical variates *ω *and *ξ*, such that *ω *= **Yu **and *ξ *= **Xv**, with the weight vectors **u***' *= (*u*_1_,..., *u*_*m*_) and **v***' *= (*v*_1_,..., *v*_*k*_). The optimal weight vectors are obtained by maximizing the correlation between the canonical variate pairs, which is also known as the canonical correlation:



The number of variables (greatly) exceeds the number of subjects, and there is often presence of multicollinearity within both sets of variables. In the regression context several penalization methods have been presented to deal with such problems and by converting the CCA problem into a regression framework, we can adapt those penalization methods for CCA. This conversion can be obtained by the two-block Mode B of Wold's original partial least squares algorithm [10,11].

Wold's algorithm is an iterative process that begins by estimating an initial canonical variate pair based on an initial guess of the weights assigned to the original variables. The objective is to maximize the canonical correlation, therefore the initial canonical variate pair *ξ *and *ω *are regressed on respectively **Y **and **X **to estimate a new set of weights. With this new set of weights, a new pair of canonical variates is determined, which are in their turn regressed on **Y **and **X**. This process is repeated until the weights converge, resulting in the first pair of maximally correlating canonical variates. Hereafter the residual matrices of **Y **and **X **are determined and the algorithm is repeated for the residual matrices to obtain the remaining pairs of canonical variates. This process can be repeated until the residual matrices contain no more information or until the decision is made that further analysis is no longer useful.

Previously we proposed penalized CCA [8] where we performed the same penalization method on both sets of variables. The elastic net [12] was used as a basis of the penalization since it solves the multicollinearity due to co-regulated and co-expressed genes, and overfitting caused by the small number of subjects and the large number of variables. Furthermore reduction of the large number of variables within the canonical variates can be obtained, such that interpretation of the results becomes easier. Elastic net combines the advantages of ridge regression (grouping effect) and the lasso [13] (built-in variable selection). Ridge regression shrinks the weights by imposing a penalty on their size, such that highly correlated variables get similar weights. The lasso is also a shrinkage method, it also shrinks the weights by imposing a penalty on their size, however where ridge regression does not shrink the coefficients to zero lasso does, resulting in variable selection. By combining the two, groups of highly correlated variables are in or out of the model. For the present situation the set of **X **variables contains usually a limited number and we therefore do not require reduction of the number of variables within the canonical variates of the **X **variables. We therefore propose an asymmetric penalization scheme: we use a ridge penalty for the set of **X **variables, thus ensuring that all **X **variables are included, and the elastic net penalty for the set of **Y **variables.

When applying the ridge penalty to the set of **X **variables and the elastic net to the set **Y **variables, the estimations of the weight vectors look as follows:





with *λ*_2*x *_the ridge penalty for the **X **variables, and *λ*_2*y *_the ridge penalty and *λ*_1*y *_the lasso penalty for the **Y **variables.

The optimal penalty parameters can be chosen with cross-validation, but because the computation time is very large due to the large number of **Y **variables, we simplified the computations. Zou and Hastie [12] suggested to fix the ridge penalty in the elastic net to infinity, resulting in univariate soft-thresholding (UST):



with *f*_+ _= *f *if *f *> 0 and *f*_+ _= 0 if *f *≤ 0. Although UST disregards the dependency between variables within the same set, the grouping effect from the ridge penalty is maintained. By employing UST for the **Y **variables we only have to choose *λ*_2*x *_and *λ*_1*y *_by cross-validation.

A second pair of canonical variates can be obtained via the residual matrices of **X **and **Y**, therefore the part of the variables that explains the first pair of canonical variates is removed from the sets.

**X**^*residual *^= **X **- *ξγ' *and **Y**^*residual *^= **Y **- *ωθ'*, where *γ *and *θ *are the vectors of linear regression weights of all X-variables on *ξ *and Y-variables on *ω*, respectively. Further canonical variate pairs can be obtained in similar way, until the residual matrix contains no more information or until the decision is made that further analysis is no longer useful.

### Cross-validation and Permutation

Optimization of the penalty parameters for each canonical variate pair is determined by *p*-fold cross-validation. The data-set is divided into *p *subgroups of patients, of which *p *- 1 subgroups form the training set and the remaining subgroup forms the validation set. The weight vectors **u **and **v **are estimated in the training set and are used to obtain the canonical correlations in the training and validation sets. This is repeated *p *times, such that each subgroup has functioned both as a validation set and part of the training set.

Instead of determining the lasso penalty(*λ*_1*y*_), it is for sake of interpretation and to reduce computation time easier to determine the number of Y-variables to be included in the final model. This approach is also used by Lê Cao *et al*. [14] and Shen and Huang [15]. The optimal number of variables were then obtained for those values of *λ*_2*x *_and *λ*_1*y *_where the mean difference between the canonical correlation of the training and validation sets is minimized:



with  and  the weight vectors estimated by the training sets **X**_-*j *_and **Y**_-*j *_in which subset *j *was deleted.

If the number of variables is very large, there is a high probability that a random pair of variables has a very high correlation by chance. Since the canonical correlation is at least as large as the largest correlation between any two variables from the two sets, canonical correlations are often very large even when correlations are zero in the population. To identify a canonical correlation that is large by chance only, we performed a permutation-analysis on the validation sets. We permuted the canonical variate *ξ *and kept the canonical variate *ω *and then determined the difference between the canonical correlation of the training and the permuted validation sets, this was compared with the difference between the canonical correlation of the training and of the non-permuted validation sets. In the permuted validation sets the variates will have zero correlation, while this is not the case for the non-permuted validation set.

## Results

### Simulations

We simulated data of 50 individuals of a pathway consisting of 50 standard normally distributed **X**-variables, whose covariances were determined by two weakly correlated components (*r *= 0.1). The first 25 **X**-variables were correlated with the first component and the other 25 **X**-variables were correlated with the second component: all correlations of the **X**-variables with the two components were sampled from the *beta*(0.5, 0.5) distribution. In addition, 999 **Y**-variables were sampled for the 50 individuals from the multivariate normal distribution with mean zero and identity covariance matrix. Next, one randomly selected variable from the **X**-set of pathway variables was removed and put in the set of **Y**-variables. This process simulated a situation where we consider 1, 000 variables as candidates for a role in the pathway that already consists of 49 variables, and with only one variable that is truly part of the pathway. In such a case we considered 49, 000 correlations of which 48, 951 (99.9%) were expected to be zero. We performed penalized canonical correlation analysis using ridge penalization for the 49 **X**-set of variables in the pathway and using the soft-threshold penalty for the 1, 000 variables in the **Y**-set. Optimal values of the two penalty parameters (*λ*_2*x *_and *λ*_1*y*_) were estimated by 10-fold cross-validation, and we selected the values minimizing the average absolute difference between the canonical correlations of the 10 training-sets and the validation-sets. All simulations were repeated 100 times, and we counted the number of simulations in which the correct variable (i.e. the **X**-variable that was put in the **Y**-set) was selected in the first pair of canonical variates.

**Figure 1:**
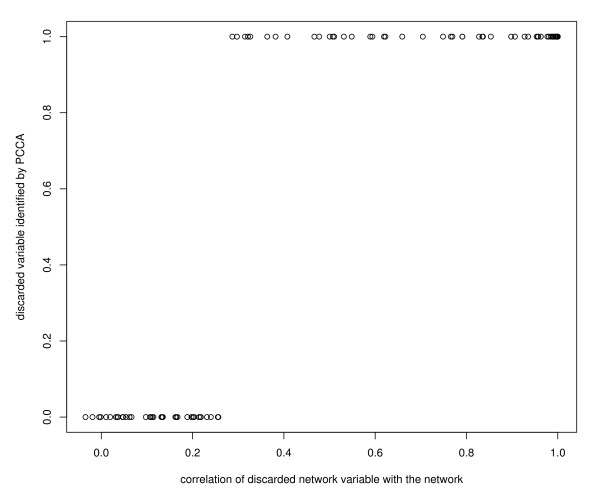
**Detection of the discarded pathway variable versus its multiple correlation with the remaining variables in the pathway**. Indication of the relation between the likelihood of identifying correctly a discarded pathway variable and the size of the multiple correlation of the discarded pathway variable with the remaining pathway variables.

In a typical simulation the absolute correlations of the **X**-variables in the pathway varied between zero and unity with mean 0.25, and the absolute correlations between any X-variable and any Y-variable varied between zero and 0.6 with mean 0.1. Since the canonical correlation is at least as large as the largest bivariate correlation it was not surprising that the probability that the discarded pathway variable was identified in the first pair of canonical variates, depended on its correlation with the other pathway variables. This relation is illustrated in Figure 1: if the correlation was larger than 0.3 the discarded variable was correctly identified.

### Glioma pathway in Glioblastoma Samples

**Figure 2:**
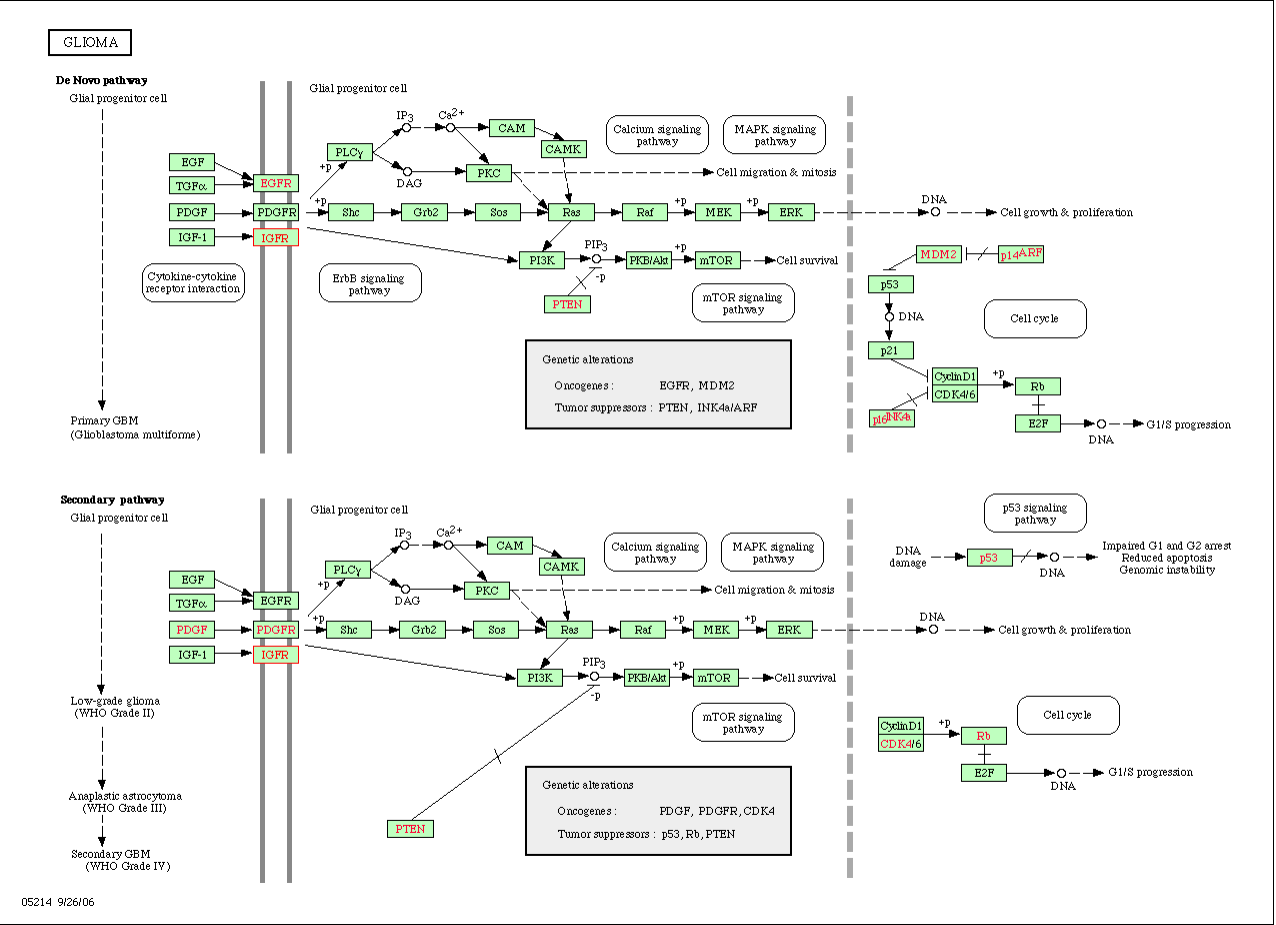
**Glioma pathway (from KEGG, November 2008)**. Graphical description of the Glioma pathway as given by the Kyoto Encyclopedia of Genes and Genomes.

**Figure 3:**
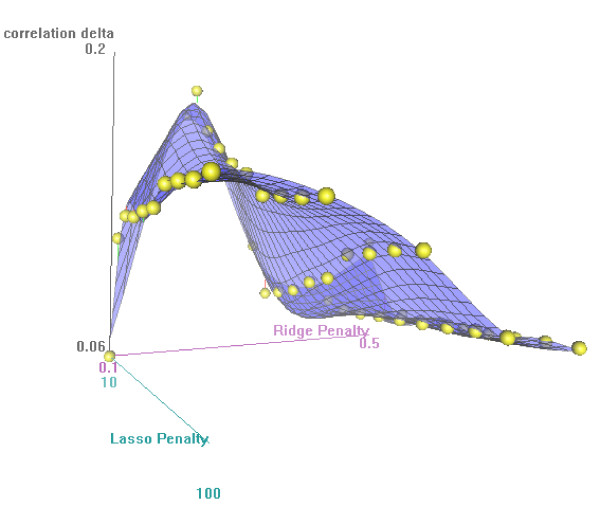
**Cross-validation criterion**. Difference between the canonical correlation of the training and validation set as a function of the ridge and lasso penalties.

**Figure 4:**
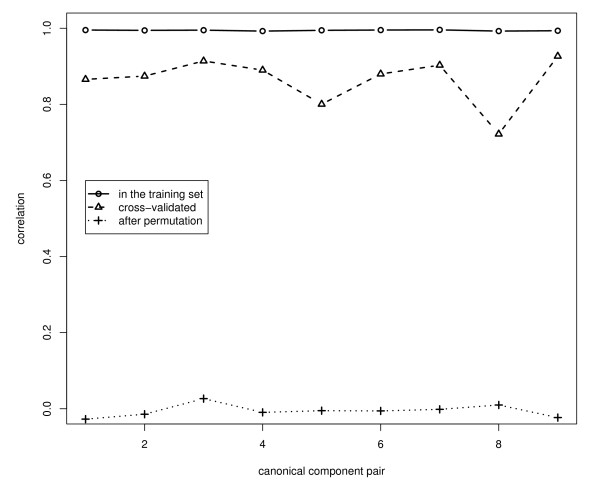
**First nine canonical correlations**. Canonical correlations in the training data set, in the validation data set and after permutations.

As an example we analyzed the expression of 12,209 genes in 45 patients with glioblastoma [8,16]: the data are available in the Stanford University Microarray Database [17]. In an earlier paper we identified the insulin growth factor receptor type I gene (IGF1R) as important in the development of glioblastoma, and therefore we concentrated on the Glioma-pathway which is highly depended on IGF1R as an example. According to KEGG (November 2008) this pathway consists of 57 genes (see Figure 2) and 55 of these were present in the set of genes available. The **X**-set consisted therefore of the expression values of the 45 patients on the 55 genes and the **Y**-set consisted of the expression values of the 45 patients of all 12, 154 other genes. Absolute correlations between the expression values of the 55 genes in the Glioma-pathway varied between zero and 0.81 with mean 0.17, and the absolute correlations between any of the 55 Glioma-pathway genes and any of the remaining 12, 154 gene expression variables varied between zero and 0.89 with mean 0.19. Principal components analysis of **X **indicated 16 components with eigenvalue > 1. We calculated nine pairs of canonical variates with PCCA and we used nine-fold cross-validation to estimate the optimal penalty-values (see Figure 3 for the first pair of canonical components). The averaged canonical correlations in the training-sets, in the validation-sets and after permutations are given in Figure 4: the canonical correlations after cross-validation were slightly smaller than the correlations in the training sets, but all were substantial larger than the correlations after permutations.

Cross-correlations between genes in the Glioma-pathway and canonical components of the genes not-in-the-Glioma-pathway are reported in Table 1, and cross-correlations between genes not-in-the-pathway and canonical components of the Glioma-pathway are reported in Table 2: in the latter table we only report those genes that have correlation > 0.9. Mapping these 32 genes on Gene Ontology using Fatigo [18] we found that this series contained a significantly (*p *= 0.00092) increased number of genes involved in immunoglobulin binding (GO: 0019865): FCGR2A, FCGR2B, and FCGR3A. IgG binding has been implicated in brain cancer by others, eg. [19] and has therapeutic consequences [20,21]. Other genes are known to be involved in glioma cells function [22-25].

**Table 1:** Cross-correlations of pathway genes with canonical variates of the remaining genes.

	**C1**	**C2**	**C3**	**C4**	**C5**	**C6**	**C7**	**C8**	**C9**
AKT1	-0.15	-0.06	0.30	0.72	0.21	0.06	-0.12	0.00	0.16
AKT2	-0.03	-0.18	0.08	0.46	0.25	-0.24	-0.05	-0.24	0.17
ARAF	-0.53	0.28	0.23	-0.01	0.12	-0.07	0.32	-0.14	0.05
CCND1	-0.16	-0.28	0.14	-0.15	0.20	0.05	-0.05	-0.36	-0.18
BRAF	0.10	0.15	0.14	0.02	0.01	-0.22	-0.13	0.17	-0.25
CAMK2A	0.89	0.00	0.08	-0.09	-0.02	0.02	0.04	0.03	0.07
CAMK2D	-0.16	0.23	0.26	-0.01	0.39	0.05	-0.08	-0.04	0.26
CAMK2G	0.44	-0.16	-0.27	0.12	-0.18	0.05	-0.20	0.12	-0.12
CDK6	-0.28	-0.09	0.31	0.06	0.51	0.12	-0.18	-0.19	0.14
CDKN1A	-0.10	0.63	0.04	0.03	0.52	0.13	0.13	0.09	-0.10
CDKN2A	-0.21	0.74	0.29	-0.22	0.11	0.04	-0.03	0.02	-0.10
E2F1	-0.11	-0.24	0.87	0.01	-0.11	0.01	-0.03	-0.01	-0.02
E2F3	-0.21	-0.16	0.61	0.22	-0.18	-0.02	0.23	0.04	-0.12
EGF	-0.16	0.38	-0.31	-0.16	0.06	-0.04	0.06	0.07	-0.13
EGFR	-0.17	-0.38	0.06	0.04	0.11	-0.16	0.02	0.43	0.32
PTK2B	-0.06	0.15	-0.09	0.71	0.03	0.08	0.11	0.18	-0.01
FRAP1	0.33	-0.13	0.05	0.07	-0.28	0.05	0.10	0.51	-0.14
GRB2	0.01	0.10	-0.02	0.30	0.17	-0.12	0.04	-0.14	0.63
HRAS	0.28	0.22	0.03	-0.40	0.26	-0.25	-0.06	0.28	0.04
IGF1	-0.01	0.73	-0.17	-0.14	-0.05	0.11	-0.07	0.30	0.14
IGF1R	-0.16	-0.39	-0.13	-0.01	0.12	0.74	-0.04	-0.18	-0.05
KRAS2	0.09	0.08	0.09	-0.02	-0.01	-0.41	0.11	0.13	-0.07
MDM2	-0.18	0.24	0.10	-0.04	0.11	0.01	0.61	-0.01	-0.12
PDGFA	-0.05	0.49	0.20	-0.23	0.61	0.10	0.04	-0.08	0.19
PDGFB	0.24	0.24	0.13	-0.01	0.19	0.05	-0.11	-0.02	0.02
PDGFRA	-0.01	-0.36	0.26	0.04	-0.05	-0.05	0.12	-0.25	-0.24
PDGFRB	-0.03	0.35	0.15	0.39	0.27	0.11	-0.12	0.21	0.13
PIK3CA	-0.19	-0.23	-0.09	0.14	0.33	-0.39	0.06	0.05	-0.21
PIK3CB	0.25	-0.01	-0.12	0.29	-0.10	0.07	0.05	0.08	0.20
PIK3CG	0.00	0.24	0.08	-0.10	0.17	0.16	0.00	0.12	0.15
PIK3R1	0.46	-0.26	-0.25	-0.02	-0.29	-0.02	-0.03	0.24	0.07
PLCG1	-0.18	-0.33	0.40	-0.06	0.15	0.19	-0.32	0.35	-0.06
PLRG1	0.00	-0.30	-0.17	0.07	0.15	-0.31	0.15	0.02	-0.12
PRKCA	0.23	-0.29	-0.25	0.61	0.17	-0.07	0.02	-0.18	-0.21
PRKCB1	0.91	0.19	-0.15	0.09	0.08	0.10	0.00	-0.04	0.07
PRKCD	0.20	0.80	-0.13	-0.07	-0.07	0.00	0.02	0.09	-0.09
PRKCG	0.83	0.26	-0.10	0.01	-0.05	0.00	-0.01	0.01	-0.02
PRKCI	0.31	0.11	0.31	0.07	-0.05	-0.11	0.02	0.36	-0.24
MAPK1	-0.02	-0.21	-0.24	0.23	-0.13	-0.13	-0.18	-0.06	0.16
MAP2K1	0.36	0.05	-0.05	-0.40	-0.09	-0.02	0.18	-0.29	-0.11
MAP2K2	-0.21	0.20	0.14	0.04	0.27	0.25	0.03	0.25	0.33
PTEN	0.13	-0.01	-0.48	0.00	0.13	0.14	-0.09	-0.25	-0.12
RAF1	-0.31	-0.29	-0.06	0.06	-0.39	-0.12	-0.12	-0.17	0.10
RB1	-0.47	0.36	0.18	-0.01	0.27	-0.05	0.06	-0.03	0.01
SHC1	-0.29	0.58	0.20	0.11	0.38	0.12	-0.11	-0.03	-0.02
SOS1	0.16	-0.20	-0.14	0.18	0.02	-0.11	0.12	-0.13	-0.05
SOS2	-0.37	-0.20	-0.26	0.02	0.04	0.03	-0.04	-0.21	-0.07
TGFA	0.22	0.13	-0.23	0.05	0.44	-0.23	0.31	-0.31	0.13
TP53	-0.40	0.20	0.35	0.02	0.23	-0.06	-0.16	-0.10	0.02
SLC4A4	0.30	-0.34	-0.37	0.11	0.12	-0.11	-0.16	0.60	0.00
AKT3	0.40	-0.37	-0.30	0.21	-0.13	0.04	-0.12	0.00	0.10
RASSF1	-0.32	0.53	0.17	-0.36	0.11	-0.07	0.04	0.12	-0.24
SHC2	0.04	-0.49	-0.06	-0.10	0.08	-0.02	-0.08	-0.22	-0.19
SHC3	0.38	0.14	0.04	0.04	0.28	0.07	0.14	0.15	0.00
RaLP	0.08	0.12	-0.24	-0.03	0.42	0.30	0.10	0.08	-0.08

**Table 2:** Cross-correlations of remaining genes with canonical variates of the pathway genes.

	**C1**	**C2**	**C3**	**C4**	**C5**	**C6**	**C7**	**C8**	**C9**
C2	-0.11	0.93	-0.03	-0.01	-0.03	0.08	-0.08	0.07	-0.02
CD68	-0.26	0.92	-0.03	0.05	-0.13	0.00	-0.04	-0.11	-0.03
CENPF	-0.26	-0.12	0.92	0.02	-0.02	0.06	0.00	0.05	-0.03
CLTB	0.91	0.01	-0.02	0.17	0.09	0.01	-0.06	-0.10	-0.08
SERPINB1	-0.11	0.91	0.02	-0.01	0.14	0.04	0.05	-0.16	-0.07
EPB49	0.90	0.06	-0.09	-0.02	0.01	0.14	0.04	0.09	0.08
FCGR2A	-0.28	0.92	-0.01	-0.09	0.00	0.00	0.01	-0.04	-0.06
FCGR2B	-0.27	0.93	-0.03	-0.02	0.01	-0.01	-0.04	0.03	0.03
FCGR3A	-0.26	0.91	0.10	0.04	-0.09	0.06	-0.01	0.02	-0.01
CXCL2	0.09	0.91	0.03	0.22	-0.03	-0.16	-0.03	0.08	-0.01
LYN	-0.20	0.93	0.04	0.11	0.05	0.03	-0.01	0.02	0.04
NEF3	0.93	0.14	-0.03	-0.15	0.01	0.02	0.01	-0.08	0.01
NEFL	0.94	-0.01	0.01	0.01	0.00	0.03	0.08	-0.10	-0.01
NRGN	0.93	0.00	0.16	-0.02	-0.02	0.01	0.01	0.02	0.00
RAB3A	0.92	-0.20	0.07	0.11	0.04	-0.08	-0.04	-0.06	0.03
SNCG	0.91	0.03	-0.12	-0.15	-0.06	0.17	0.07	0.04	0.05
STK6	-0.21	0.01	0.93	0.01	0.00	-0.01	0.04	0.16	-0.03
TNFAIP2	-0.08	0.95	-0.04	0.00	0.10	-0.03	-0.02	-0.01	-0.02
VSNL1	0.96	0.04	0.03	0.03	0.01	0.02	-0.04	-0.02	-0.02
VAMP8	-0.20	0.94	0.01	0.04	-0.07	-0.03	0.09	-0.06	-0.02
CCNB2	-0.26	-0.18	0.90	0.04	0.03	-0.08	0.07	-0.04	0.01
PHYHIP	0.94	-0.04	-0.07	-0.03	0.01	-0.02	-0.06	0.02	-0.10
TACC3	-0.30	0.04	0.90	-0.11	-0.07	-0.10	-0.06	0.00	0.01
NPC2	-0.30	0.92	-0.11	-0.01	-0.01	-0.01	-0.01	0.01	-0.03
UBE2C	-0.25	-0.20	0.93	0.01	-0.01	0.05	-0.05	0.04	-0.01
SULT4A1	0.95	0.06	0.03	-0.05	-0.05	-0.02	-0.05	0.04	-0.02
OSTF1	0.16	0.91	0.03	0.02	0.02	-0.10	-0.05	0.09	0.03
MS4A4A	-0.20	0.90	-0.06	0.06	-0.23	0.00	-0.03	0.00	-0.06
SLC17A7	0.92	-0.03	0.14	-0.02	-0.09	0.10	0.10	0.06	-0.02
NAPB	0.95	0.02	0.00	0.04	0.04	-0.02	0.02	0.15	0.01
FLJ14503	0.93	-0.01	-0.04	0.10	0.07	-0.03	0.05	-0.10	0.06
LOC441168	-0.17	0.91	-0.01	-0.01	-0.02	0.01	0.03	-0.04	0.15

## Discussion

We generalized penalized canonical correlation analysis (PCCA) for a situation where different penalty-schemes are preferred for the two data-sets involved in PCCA. We focussed this application of PCCA on checking the completeness of known metabolic pathways using microarray gene-expression measurements, and to identify candidate genes for incorporation in the pathway. We used Wold's [10] method for calculation of the canonical variates, because this algorithm was easily extended with penalty terms to deal with the situation where the number of variables is larger than the number of samples, or to perform variable selection. We applied ridge penalization to the regression of pathway genes on canonical variates of the non-pathway genes, and the elastic net to the regression of non-pathway genes on the canonical variates of the pathway genes. With ridge penalization we dealt with the multicollinearity in the data-set and with the elastic net we also performed variable selection.

We performed a small simulation to illustrate the model's capability to identify new candidates genes to incorporate in the pathway. We simulated a pathway of 50 genes and 999 unrelated genes measured in 50 patients. We discarded one pathway-gene and combined it with the set of unrelated genes, and then we performed PCCA. The probability to identify the discarded gene depended on the size of the multiple correlation of the discarded gene with the 49 other genes in the pathway, in our simulation we identified the gene correctly if the correlation was larger than 0.3.

We applied the method to a gene-expression microarray data set of 12, 209 genes measured in 45 patients with glioblastoma [16], and we considered genes to incorporate in the Glioma-pathway involved in the development of glioblastoma. We identified a large set of candidate genes that had very large correlations (> 0.9) with the canonical components of the Glioma-pathway genes. None of these were known to be associated with the glioblastoma according to PubMed, but some are known to be involved in glioma cells function [22-25].

In this application we start from existing knowledge on metabolic pathways available in knowledgebases such as KEGG [2] or Reactome [26]. We used the pathway uncritically, as it was present in the database. Reliability and completeness of pathways may vary between different knowledgebases [27], and this will influence the results to some degree. In the present case, the Glioma pathway was present in KEGG only. Although prior knowledge is often less systematic for clinical research, similar methods may be used in modeling clinical data [28].

There are few alternative methods for finding new genes as candidates for inclusion in an existing pathway. The most simple approach would be to calculate the bivariate correlations between all genes in the pathway and all genes not in the pathway, or to develop independent regression models for each gene in the pathway as a function of all genes not in the pathway. In our example of the glioma-pathway this yielded 55 × 12, 154 = 668, 470 correlations of which 1, 008 were significant after Bonferroni multiple testing correction, and this concerned 696 different genes. Application of independent regression model in combination with elastic net penalization yielded 3, 294 regression weights that were ≠ 0 and this concerned 2, 724 different genes. Thus these approaches provided far too many candidate genes in comparison to the penalized CCA approach.

## Conclusion

In conclusion, we developed penalized canonical correlation analysis (PCCA) for assessing the multivariate association of the expressions of genes in a known metabolic pathway with the expressions of a large set of candidate genes to be involved in that pathway. We used asymmetric Ridge and elastic penalization to handle the situation where the number of variables is larger than the number of samples and to perform variable selection. In a simulation study the method showed that it was capable to select relevant variables, and we applied it to microarray data of over 12, 000 genes in 45 patients with glioblastoma.

## Authors' contributions

SW developed the algorithms, carried out the statistical analyses, and drafted the manuscript, AHZ conceived the statistical models, carried out the statistical analyses and drafted the manuscript. Both authors read and approved the final munscript. SW was supported by the Netherlands Bioinformatics Centre (NBIC). AHZ was supported by grant no. 37697 from the European Union in the FP6 project GeneCure.
